# Multi-Domain Entropy-Random Forest Method for the Fusion Diagnosis of Inter-Shaft Bearing Faults with Acoustic Emission Signals

**DOI:** 10.3390/e22010057

**Published:** 2019-12-31

**Authors:** Jing Tian, Lili Liu, Fengling Zhang, Yanting Ai, Rui Wang, Chengwei Fei

**Affiliations:** 1 Liaoning Key Laboratory of Advanced Test Technology for Aeronautical Propulsion System, Shenyang Aerospace University, Shenyang 110136, China; jingtian@sau.edu.cn (J.T.); 20123290@sau.edu.cn (L.L.); fling707@buaa.edu.cn (F.Z.);; 2 Department of Power and Energy, Northwestern Polytechnical University, Xi’an 710129, China; wangrui2019@mail.nwpu.edu.cn; 3 Department of Aeronautics and Astronautics, Fudan University, Shanghai 200433, China

**Keywords:** multi-domain entropy, information entropy, random forest, inter-shaft bearing, fault diagnosis

## Abstract

Inter-shaft bearing as a key component of turbomachinery is a major source of catastrophic accidents. Due to the requirement of high sampling frequency and high sensitivity to impact signals, AE (Acoustic Emission) signals are widely applied to monitor and diagnose inter-shaft bearing faults. With respect to the nonstationary and nonlinear of inter-shaft bearing AE signals, this paper presents a novel fault diagnosis method of inter-shaft bearing called the multi-domain entropy-random forest (MDERF) method by fusing multi-domain entropy and random forest. Firstly, the simulation test of inter-shaft bearing faults is conducted to simulate the typical fault modes of inter-shaft bearing and collect the data of AE signals. Secondly, multi-domain entropy is proposed as a feature extraction approach to extract the four entropies of AE signal. Finally, the samples in the built set are divided into two subsets to train and establish the random forest model of bearing fault diagnosis, respectively. The effectiveness and generalization ability of the developed model are verified based on the other experimental data. The proposed fault diagnosis method is validated to hold good generalization ability and high diagnostic accuracy (~0.9375) without over-fitting phenomenon in the fault diagnosis of bearing shaft.

## 1. Introduction

Inter-shaft bearing operation between high- and low-pressure rotors is a key component of aeroengines. The failure of inter-shaft bearing can have a catastrophic effect on aeroengines. Identifying and diagnosing inter-shaft faults of aeroengines early and accurately are promising to avoid major accidents, and thus have significant economic benefit and engineering signification [1].

Nowadays, there are many ways to monitor fault signals of rolling bearings, such as noise signals, vibration signals, and AE (Acoustic Emission) signals. Noise signals often contain more environmental noise. It is difficult to identify the fault signals. Hence, it is often used in combination with a vibration signal in practical application. The vibration signal has become the most widely used monitoring signal due to its easy detection and intuitive signal expression. However, the inter-shaft bearing is located in engine rotor, and its vibration signal is easily affected by the connection part and the transmission part, so that the vibration signal is drowned by other noise signals. The AE signal is released outwardly in the form of a instantaneous elastic wave when the energy accumulates to a certain extent due to the unstable stress distribution inside the object when it is subjected to deformation. Common faults of rolling bearings, such as wear, deformation, and crack, will produce a large number of AE signals. As such, the AE signal is widely used in inter-shaft bearing fault diagnosis, owing to its high frequency and sensitive characteristics to impact signals [2]. Although AE signals can better avoid the interference of noise signals compared with vibration signals, the AE signal of faults is still relatively weak and contains a lot of mechanical noise due to a complicated signal transmission path. Therefore, proposing an effective signal analysis technique, improving the signal-to-noise ratio, and identifying the fault accurately, is always the hot direction of experts and scholars. In recent years, a variety of effective fault diagnosis techniques have been developed based on information fusion theory and pattern recognition methods. Hsieh et al. used the combination of empirical mode decomposition (EMD) and multi-scale information entropy to accurately identify many high-speed rotor faults, such as the imbalance, misalignment, and poor lubrication [3]. For the imbalance, cracks of motor rotors, and single, coupling faults of bearings, Romero, et al. revealed that the fuzzy logic reasoning method could be precise in classifying and identifying the information entropy of different faults, and developed an on-line monitoring system with the above theory [4]. Yu et al. proposed a motor rolling bearing fault diagnosis method based on pattern spectrum entropy and proximal support vector machine (PSVM) [5]. Ai et al. introduced the fusion information entropy distance method for the fault diagnosis of rolling bearing based on wavelet spectral entropy, singular spectral entropy, power spectral entropy, and wavelet spectral entropy of the AE and vibration signals [6]. Based on the combination of singular value decomposition and information entropy, Hernandez et al. extracted the fault features of faulty rotors and bearings to accurately identify faults [7]. Information entropy is a measure of information uncertainty. The larger the value is, the higher the system complexity is. As some faults have similar signal characteristics, information entropy can only measure their complexity and perform preliminary noise reduction and classification but cannot accurately classify. Recently, information entropy has been widely applied as the feature extraction method of fault diagnosis [8,9,10,11,12,13,14]. The purpose of fault diagnosis is to predict the discrete values at different fault states of a diagnostic object. It is a classification task; the core issue is classifier design. Random forest is a combined classification algorithm of a decision-tree based on the stochastic statistical theory and belongs to supervised learning method. This algorithm is a nonlinear modeling tool which has the advantages of fast calculation speed, high classification accuracy, and extensive generalization ability. When learning sample features are obvious, it can obtain better classification accuracy and robustness. It has been applied in many fields, such as finance and biology, and achieved good classification results [15]. Gómez-Peñate et al. presented the design of a *H* sliding mode and an unknown input observer for Takagi-Sugeno (TS) systems. Contrary to the common approaches of considering exact premise variables, this work deals with the problem of inexact measurements of the premise variables. The method is robust to disturbances, sensor noise, and uncertainty on the premise variables [16]. Kobayashi et al. proposed a new fault auto-detection method by which the signals measured by an accelerometer located at a far point from the diagnosed bearing can be used to detect the bearing faults automatically [17]. Santos-Ruiz et al. described a data-driven system based on PCA (Principal Component Analysis) to detect and quantify fluid leaks in an experimental pipeline and use a dynamic PCA implementation (DPCA) to capture the process dynamics [18].

To make up the traditional information entropy method in extracting strong non-stationary inter-shaft bearing fault signal, this paper establishes a fusion of multiple information entropies with many analysis domain characteristics, namely multi-domain entropy. This method is based on the theory of information entropy fault diagnosis and presents a fault diagnosis method of multi-domain entropy-random forest by integrating the advantages of multi-domain entropy and random forest. The simulation experiment of four typical faults is conducted on inter-shaft bearing fault simulation rig. The multi-domain entropy of fault for the AE signal is extracted to build inter-shaft bearing fault feature vector samples. Random forest is generated by fault sample data, and these data are adopted to test the accuracy and generalization ability of random forest diagnosis and verify the effectiveness of the multi-domain entropy-random forest fault diagnosis method.

The structure of the paper is presented as follows. Four information entropies-singular spectrum entropy (SSE), power spectrum entropy (PSE), wavelet energy spectrum entropy (WESE), and wavelet space feature spectrum entropy (WSFSE), which reflect different domains and multi-domain entropy are introduced in Section 2. The process to build and evaluate RF (Random Forest) are introduced in Section 3. In Section 4, the multi-domain entropy-random forest method is proposed. In Section 5, the rolling bearing faults simulation experiments are carried out to evaluate the present method. Finally, conclusions are given in Section 6.

## 2. Multi-Domain Entropy Approach for Feature Extraction

### 2.1. Information Entropy Theory

Information entropy is a concept used to measure information content in information theory. The more orderly a system is, the lower the information entropy is. Conversely, the more confused it is, the higher the information entropy is. Therefore, information entropy can also be said to be a measure of the systematic ordering degree [8]. The information entropy of the normal bearing is lower than the fault bearing, therefore, we can use it to evaluate the bearing working status.

Assuming the Lebesgue space *M* with the measure *μ* (*μ*(*M*) = 1), that garners by a measurable set *H*, which may be denoted as the incompatible set with a limited partitioning *A* = {*A_i_*}, i.e., $M=\overset{n}{\underset{i=1}{\cup }}A_{i}$, where *A_i_* ∩ *A_j_* = 0, *i* ≠ *j*, the information entropy of the set *A* is [9]

(1) $$S(A)=-\sum_{i=1}^{n}\mu (A_{i})\log\mu (A_{i})$$

where *μ*(*A_i_*) is the measurement of the *i*th sample *A_i_*, *i* = 1, 2, …, *n*.

In conclusion, when the rolling bearing state will be evaluated by information entropy, according to the characteristics of AE signal to choose the appropriate classification system and the corresponding measurement indicator.

### 2.2. Extraction of Information Entropy Features in Many Analytical Domains

#### 2.2.1. Time Domain Information Entropy Features

The AE signal of any measuring point is a discrete time series. By the delay embedding technique, an arbitrary AE signal {*x_i_*}(*i =* 1, 2, …, *n*) is mapped to an embedded space. *N* is the number of samples. When the space length of a modal window is *M*, and the delay constant is 1. Then the signal $\left\{x_{i}\right\}$ can be divided into N−M segment modal data to obtain a pattern matrix **A**, i.e.,

$$A=\left[\begin{array}{cccc} x_{1} & x_{2} & \cdots & x_{M}\\ x_{2} & x_{3} & \cdots & x_{M+1}\\ \vdots & \vdots & \cdots & \vdots \\ x_{N-M} & x_{N-M+1} & \cdots & x_{N} \end{array}\right]$$

In line with the singular value decomposition (SVD), the singular values $\left\{\sigma_{i}\}(1\leq i\leq M\right)$ of the matrix **A** are gained. The number of non-zero singular values reflects the number of patterns contained in each column of the matrix. The size of them reflects the proportion of the mode to the total mode. Then, in light of the thought on information entropy, the singular value is a kind of time domain division of AE signal [10]. The singular spectrum entropy (SSE) of AE signal is

(2) $$H_{t}=-\sum_{i=1}^{M}p_{i}\ln p_{i}$$

in which $p_{i}=\sigma_{i}/\sum_{i=1}^{M}\sigma_{i}$ is the ratio of the *i*th singular spectrum to the whole spectrum. The maximum singular spectrum entropy is white noise $H_{t,\max}=\log M$. According to this feature, the signal may be normalized by white noise. The SSE formula is rewritten as

(3) $$H_{t}=\frac{-\sum_{i=1}^{M}p_{i}\log p_{i}}{\log M}$$

#### 2.2.2. Frequency Domain Information Entropy Feature

When frequency signal *X*(*ω*) is the discrete Fourier transform of an AE time signal {*x_t_*}, its power spectrum is $S(\omega )=\frac{1}{2\pi N}{\left|X(\omega )\right|}^{2}$. The AE signal transformation from time domain to frequency domain obeys the conservation of energy; the relationship is as follows,

(4) $${\sum x^{2}(t)\Delta t=\sum \left|X(\omega )\right|}^{2}\Delta \omega$$

Therefore, *S* = {*S*_1_, *S*_2_, *…*, *S_N_*} may be regarded as the partition of the original signal. Then the power spectrum entropy (PSE) *H_f_* of the AE signal is defined by

(5) $$H_{f}=-\sum_{i=1}^{N}q_{i}\ln q_{i}$$

where $q_{i}=S_{i}/(\sum_{i=1}^{N}S_{i})$ is the ratio of the *i*th power spectrum to the whole spectrum. Similarly, normalized by the white noise signal [19,20,21]. The PSE of the white noise is *H_f_*_,max_ = log*N*. Then the PSE formula is rewritten as

(6) $$H_{f}=\frac{-\sum_{i=1}^{N}q_{i}\log q_{i}}{\log N}$$

#### 2.2.3. Time-Frequency Domain Information Entropy Features

Wavelet analysis is a time-frequency analysis method developed based on overcoming the Fourier transform shortcomings [22]. The AE signal function is *f*(*t*). Its limited energy is conserved for the wavelet transform by

(7) $$\left\{ \begin{array}{l} \int_{-\infty }^{+\infty }{\left|f(t)\right|}^{2}dt=\frac{1}{C_{\psi }}\int_{0}^{\infty }E(a)da\\ C_{\psi }=\int_{-\infty }^{+\infty }\frac{{\left|\psi (\omega )\right|}^{2}}{\omega }d\omega \\ E(a)=\int_{-\infty }^{+\infty }{\left|W_{f}(a,b)\right|}^{2}db \end{array}\right.$$

where *C_Ψ_* is the admissible condition of the wavelet function; *E*(*a*) the energy of the function *f*(*t*) when the scale of *f*(*t*) is *a*.

When *E* = {*E*_1_, *E*_2_, …, *E_n_*} denotes a wavelet spectrum of signal f(t) on *n* scales, the E is regarded as the partition of signal energy according to the definition of information entropy. Thus, the time-frequency domain wavelet energy spectrum entropy (WESE) $H_{\mathrm{we}}$ [23] is defined by

(8) $$H_{we}=-\sum_{i=1}^{n}s_{i}\ln s_{i}$$

where $s_{i}=E_{i}/\sum_{i=1}^{n}E_{i}$ is the ratio of the *i*th wavelet energy spectrum to the whole spectrum.

The wavelet transform is to isometrically map the one-dimensional signal into two-dimensional space. $W=\left[{\left|W_{f}(a,b)\right|}^{2}/C_{\psi }a^{2}\right]$ is the energy distribution matrix of the signal on two-dimensional wavelet space. Through the SVD of the matrix *W*, similar to SSE, the time-frequency domain wavelet space feature spectrum entropy (WSFSE) [24] $H_{ws}$ is expressed as

(9) $$H_{ws}=-\sum_{i=1}^{n}r_{i}\ln r_{i}$$

where $r_{i}=\sigma_{i}/\sum_{i=1}^{n}\sigma_{i}$ is the ratio of the *i*th feature spectrum to the whole spectrum.

The basis function formed by wavelet is a division of signal energy in scale space, which reflects the energy distribution characteristic of signal in time and frequency domain and measures the information ordering of the rolling bearing AE signal accurately.

### 2.3. Multi-Domain Entropy

From the analysis above, we can see that four information entropies—SSE, PSE, WESE, and WSFSE—could reflect the complexity of AE signal, just reflected by the different domains, in the acceleration or deceleration of bearing. Fusing the four information entropies can comprehensively analyze fault information with the AE signal. This method can improve the utilization of information and diagnose the fault in the early weak signal. In this paper, four information entropies are formed into a four-dimensional space. For the rolling bearing fault, four information entropies can be obtained. Each fault entropy band will change within a small range of values. By obtaining the mean value of each fault entropy band, the information entropy center-information entropy point. Combining four information entropy points $\left(H_{t},H_{f},H_{we},H_{ws}\right)$, one multi-domain entropy point in a four-dimensional space can be determined.

(10) $$d_{i}=\sqrt{\sum_{j=1}^{4}{\left(E_{ij}\right)}^{2}}$$

where *i* (*i* = 1, 2, 3, 4) is the information entropy category; *j* (*j* = 1, 2, 3, 4) is the sensor position.

## 3. Random Forest Method for Fault Diagnosis

Random forest method is a kind of statistical theory proposed by Breimans, which is combined with the “Bootstrap aggregating” and “random subspace” method. This method is a nonlinear modeling tool and overcomes some shortcomings: Low accuracy of single decision-tree and overfitting. Random forest method is very suitable for solving failure problems such as when priori knowledge is unclear, there is incomplete data, etc.

### 3.1. Random Forest Algorithm Building

Random forest is a classifier consisting of a collection of decision-tree classifiers. The establishment of the algorithm is divided into three steps as follows.

(1) *T* training samples are extracted from the original data set with return by Bootstrap sampling method. The number of samples is the same as the original data set [22].

Assuming that *X* is a data set containing *n* samples $\left\{x_{1},x_{2},\cdots x_{n}\right\}$, a sample $x_{i}$(i=1,2,⋯n) is extracted from the original data set *X*. And a total of *n* times is taken to combine it into a new set $X^{*}$. Then, the probability of $X^{*}$ without a sample $x_{j}$ is:

(11) $$p={\left(1-\frac{1}{n}\right)}^{n}$$

(12) $$\underset{n\to \infty }{\lim}p=\underset{n\to \infty }{\lim}{\left(1-1/n\right)}^{n}=e^{-1}\approx 0.368$$

When *n* is large enough, about 36.8% of the samples in the original data set will not be extracted. When this is the case, the decision-tree of the random forest cannot determine a local optimal solution. As such, it can effectively avoid that abnormal data appearing in the sample set, and can get a better classifier. Meanwhile, the undetected Out-Of-Bag (OOB) is used to estimate the generalization error, the correlation coefficient, and the intensity of the decision-tree. Therefore, the algorithm classification accuracy can be quantified.

(2) The *T* decision-tree models $h_{i}(X^{*},\Theta_{k})$ are built for the T training samples $X_{{}^{1}}^{*},X_{2}^{*}\cdots X_{T}^{*}$, in which i=1,2,⋯T, k=1,2,⋯.

The decision-tree model mentioned in this paper is shown in Equation (13).

(13) $$c(x_{1},x_{2},\cdots x_{n},h_{t})=\{\begin{array}{c} \mathrm{label}(h_{t})\\ c(x_{1},x_{2},\cdots x_{n},h_{t}) \end{array}\begin{array}{c} h_{t}\;\mathrm{is}\;\mathrm{the}\;\mathrm{leaf}\;\mathrm{node}\\ h_{t}\;\mathrm{is}\;\mathrm{the}\;\mathrm{inner}\;\mathrm{node} \end{array}$$

(14) $$h_{i}(X^{*},\Theta_{k})=c(x_{1},x_{2},\cdots x_{n},\mathrm{root}(h_{i}))$$

where $\mathrm{root}(h_{i})$ is the root node of decision-tree $h_{i}(X^{*},\Theta_{k})$. $c(x_{1},x_{2},\cdots x_{n},h_{t})$ is the segmentation criterion of decision-tree $h_{i}(X^{*},\Theta_{k})$. The segmentation criterion consists of a segmentation variable and segmentation predication and is measured by impurity function.

The Gini coefficient [20] reflects the inconformity probability of the category labels, in which the two samples randomly selected in the data set. The Gini coefficient is proportional to the impurity level. The optimal segmentation is to find the largest segmentation of the Gini coefficient, as follows:

(15) $$Gini(t)=1-\sum_{j=1}^{J}{\left\{p(j|t)\right\}}^{2}$$

where p(j|t) is the *j*th category probability in the node *t*, namely it is the ratio of *j*th category to sample label total *J*.

Before selecting attributes on each non-leaf-node, randomly selecting *m* attributes from *M* attributes as the classification attribute set of the current node. This is done according to the empirical formula [25] given by Liaw, usually taken as $m=\mathrm{int}(\sqrt{M})$, where int is the rounding function. The node is spited by the best division mode of *m* attributes, by which a complete decision-tree is built. The growth of each decision-tree is not pruned, until the leaf-node growing.

The random forest, generated by *T* decision-trees, is used to classify the test sample. Each tree has a voting right to decide the classification result. Summarizing the decision-tree output categories, the most classified categories are the final classification result. The classification decision model H(x) is shown as Equation (16).

(16) $$H(x)=\begin{array}{cc} \arg & \underset{Y}{\max} \end{array}\sum_{i=1}^{T}I(h_{i}(X^{*},\Theta )=Y)$$

where $h_{i}(X^{*},\Theta )$ is the single decision-tree; *Y* is the output tag variable; *I*(*) is the indicative function.

**Figure 1:**
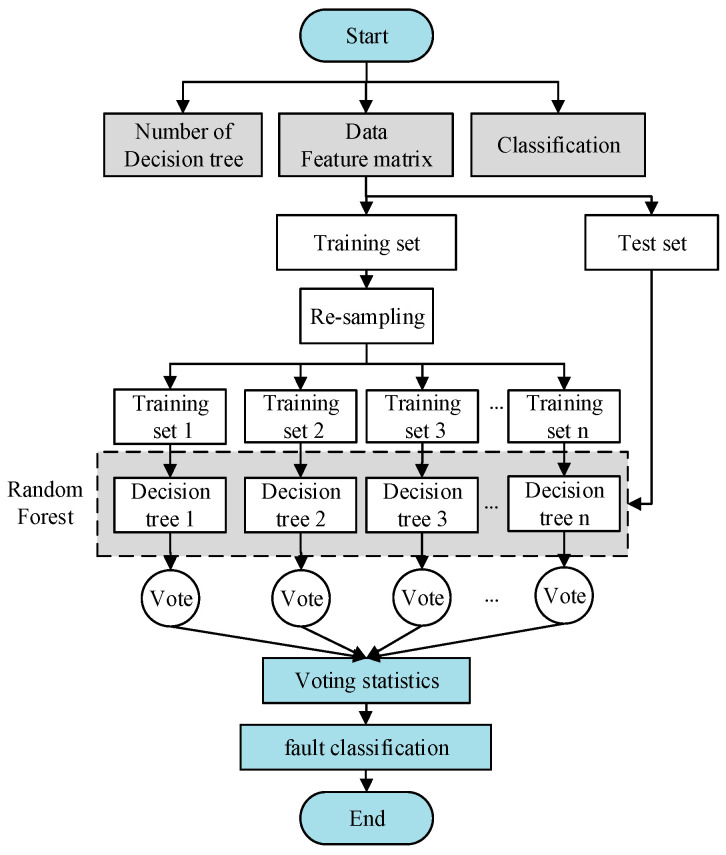
Flow chart of random forests.

The establishment and testing of the random forest is shown in Figure 1.

### 3.2. Random Forest Performance Evaluation

Generalization ability is the ability of the learning model to predict other variables, the learning model is obtained based on the training sample. The generalization error is an indicator of responding to the generalization ability. Its size has a closed relationship with the learning performance of the machine. The smaller the generalization error, the better the machine learning performance. Conversely, the greater the worse.

Random forest (RF) uses the OOB (out-of-bag) mode [26] to estimate the generalization error of the classification algorithm PE*, strength *s*, and correlation coefficient *ρ*. The error rate of the decision-tree is counted by OOB data, specifying each decision-tree as a unit. Then, the average of all decision-tree error rates is taken as an estimate of generalization error. Breiman proves through experiment that OOB error is an unbiased estimate. With the increase of the decision-tree classification model, all the sequences $\Theta_{1},\ldots \Theta_{n},PE*$ converge to $P_{X,Y}\left\{P_{\Theta }\left[h\left(X,\Theta \right)=Y\right]-max_{j\neq k}P_{\Theta }\left[h\left(X,\Theta \right)=j\right]<0\right\}$ almost everywhere.

## 4. Multi-Domain Entropy-Random Forest Method for Fault Diagnosis

**Figure 2:**
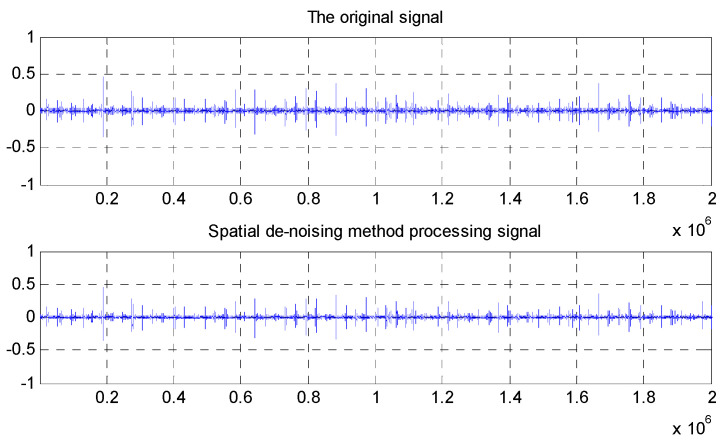
The comparison between AE (Acoustic Emission) signal before and after the preprocessing.

**Figure 3:**
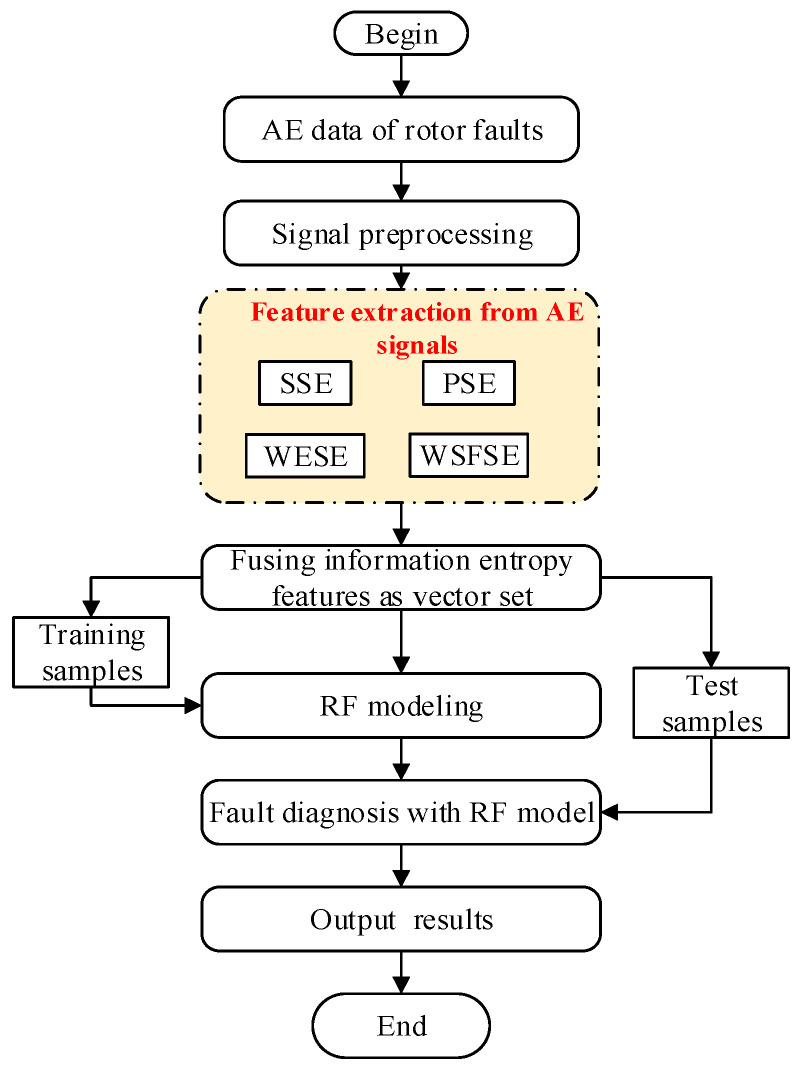
Fault diagnosis model of rotor with the multi-domain entropy-random forest (MDERF) method.

This paper proposes a fault diagnosis method for inter-shaft bearing; that is multi-domain entropy-random forest method, based on the theory of information entropy and random forest. Firstly, we establish the extraction algorithms of SSE, PSE, WESE, and WSFSE, based on the information entropy theory and the non-stationary signal processing method. At the same time, the spatial de-noising method was used to filter and reduce the noise of the collected AE signals. The comparison between an AE signal before and after the preprocessing is shown in Figure 2. Secondly, four information entropies of the fault signals are extracted to fuse, and a fault feature vector set of inner-shaft bearing is formed, after which the training samples and test samples of bearing are established. Then, training samples are used to generate random forest, and selecting the random forest attribute for training. It establishes a fault diagnosis model for random forest. Finally, a test sample is used to verify the trained random forest model. The multi-domain entropy-random forest model, proposed in this paper, is shown in Figure 3.

## 5. Fusion Diagnosis of Inter-Shaft Bearing Faults

### 5.1. Rolling Bearing Faults Simulation Experiment

The state of the inter-shaft bearing is different at different speeds, and different information is included in the raising and lowering speed. SSE, PSE, WESE, and WSFSE of the inter-shaft bearing AE signal reflect the fault state from the time, frequency, and time-frequency domain. Integrating the above four information entropies can more comprehensively and accurately assess the state of the inter-shaft bearing. To verify the effectiveness and practicability of the multi-domain entropy-random forest fault diagnosis method, a fault simulation experiment of the cylindrical roller bearing model NU202 is carried out. It simulates four typical faults (ball fault, inner race fault, outer race fault, and normal) under multi-rotation speeds and multi-measuring points and acquires an AE signal.

**Figure 4:**
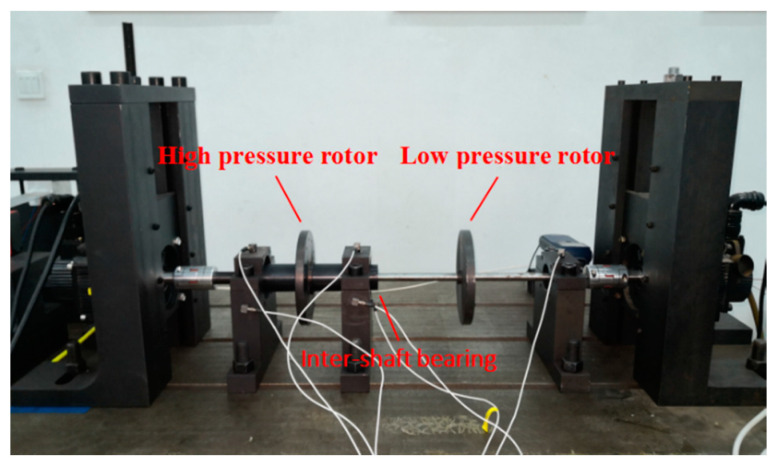
Rolling bearing fault simulation test system.

**Figure 5:**
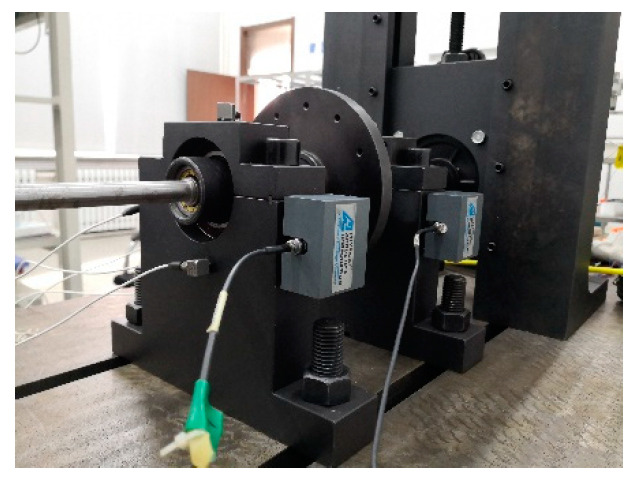
Acoustic emission sensor location.

The test system is shown in Figure 4; the double rotor test stand with inter-shaft bearing can simulate the fulcrum bearing and inter-shaft bearing failure status of the aeroengine. The acoustic emission system of PAC is adopted to collect and analysis AE signal. Four sensors are installed on the casing and bearing, as shown in Figure 5. The speed range of each fault is limited by the interval speed of 100 rpm from 800 rpm to 2000 rpm. The sampling frequency is set at 1000 KHz, and is therefore gained by 52 groups of AE signals. SSE, PSE, WESE, and WSFSE of all the rotational speeds and measurement points of AE signals are used to fuse, and the fused information entropy points are calculated according to Equation (10).

### 5.2. Extraction of Many Information Entropy Features of AE Signals

**Figure 6:**
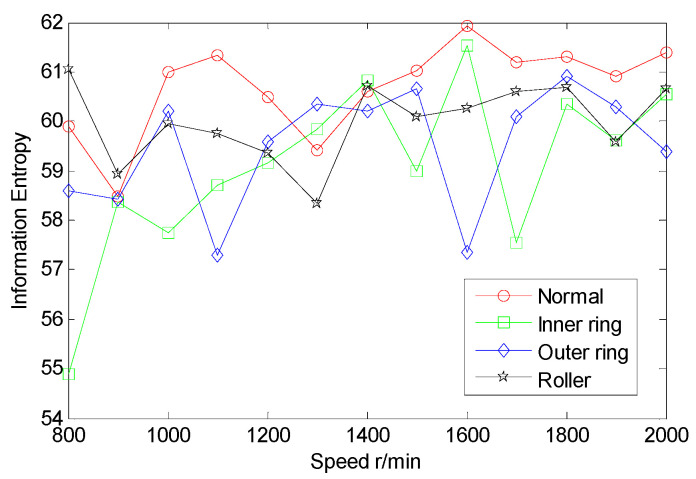
SSE curves of inter-shaft bearing four status.

**Figure 7:**
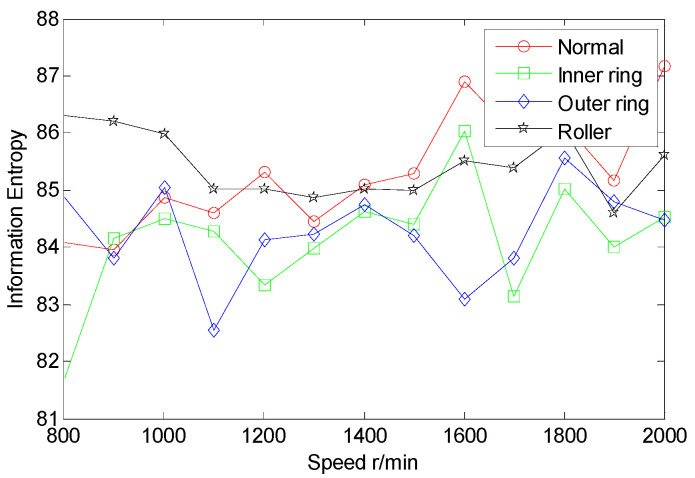
Power spectrum entropy (PSE) curves of inter-shaft bearing four status.

**Figure 8:**
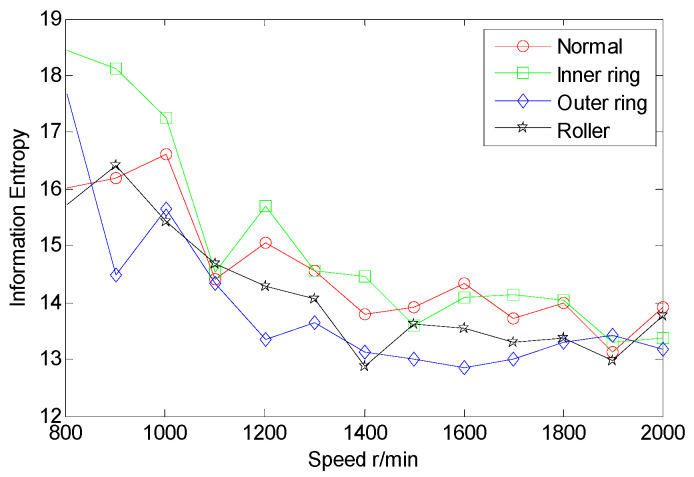
Wavelet energy spectrum (WES) curves of inter-shaft bearing four status.

**Figure 9:**
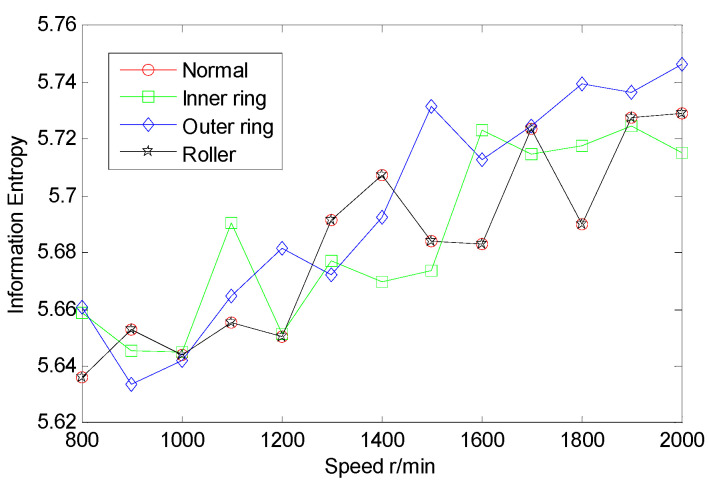
Wavelet space feature spectrum entropy (WSFSE) curves of inter-shaft bearing four status.

From the inter-shaft bearing fault simulation experiment, AE signal samples of ball fault, inner race fault, outer race fault, and normal statues are collected. SSE, PSE, WESE, and WSFSE of AE signals of the four faults are structured in terms of Equations (1) and (8). Each of the curves in Figure 6 is the SSE of a fault at multiple rotational speeds. By comparing SSE curves of the four statues, the four curves cross each other seriously, and the fault data are poorly separable. Similarly, from Figure 7, Figure 8 and Figure 9, PSE, WESE, and WSFSE curves also cross, and are not suitable for fault features alone.

### 5.3. Extraction of Multi-Domain Entropy Features of AE Signals

**Figure 10:**
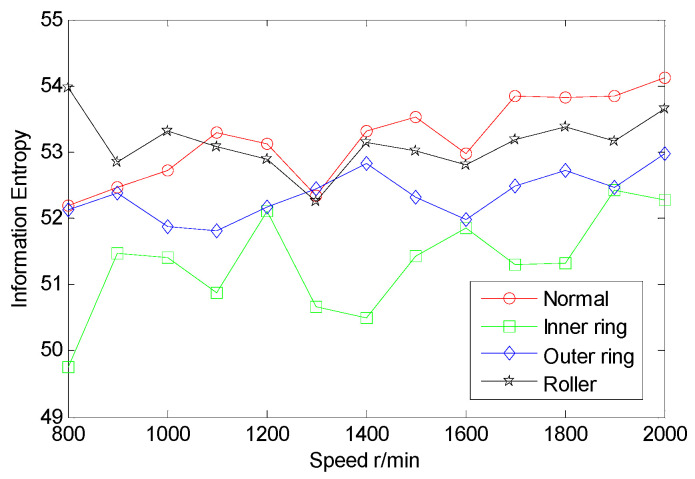
Multi-domain entropy point (MDEP) curves of inter-shaft bearing four status.

The multi-domain entropy points (MDEP) of AE signals are structured in terms of Equation (10). Each of the curves in Figure 10 is the MDEP of a fault at multiple rotational speeds. By comparing entropy point curves of the four statues, the four curves cross less, and they are basically separated. The fault data are well separable, and are suitable for fault features.

### 5.4. Fusion Diagnosis of Inter-Shaft Bearing Faults with MDERF Method

#### 5.4.1. MDERF Modeling

For each fault mode, 28 tests are conducted at each rotational speed. In total, 112 fault samples are collected, from which 20 samples of each fault are regarded as one set of training samples for MDERF modeling and the remaining eight groups of samples for each fault are termed as one set of testing samples to test the MDERF model. Herein, each sample is one feature vector of MDEP at different rotational speeds. In the MDERF model, the RF is consisted of 500 decision-making trees. Selected training samples with the MDEP are listed in Table 1.

#### 5.4.2. Validation of MDERF Method

##### Learning Ability Verification

**Figure 11:**
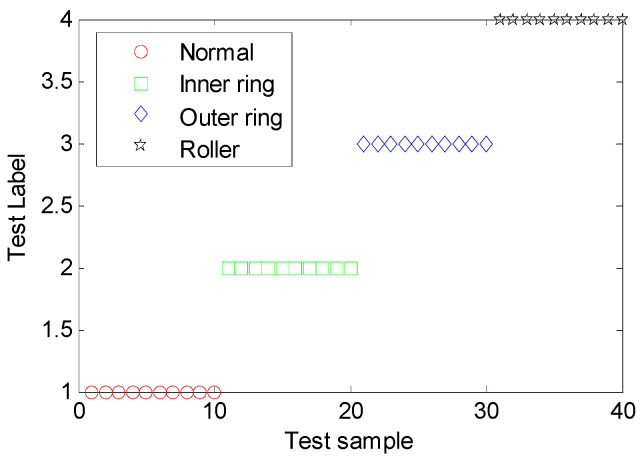
Diagnostic results of training sample on inter-shaft bearing faults.

To check the modeling accuracy and generalization of the built MDERF model, we selected 10 samples for each fault from training samples to form the set of 40 samples and then tested the established MDERF fault diagnosis model. The diagnostic results are shown in Figure 11 and Table 2. As illustrated in Figure 11 and Table 2, the MDERF model completely classifies the training samples with 100% accuracy and does not have an over-fit phenomena.

##### Generalization Ability Verification

**Figure 12:**
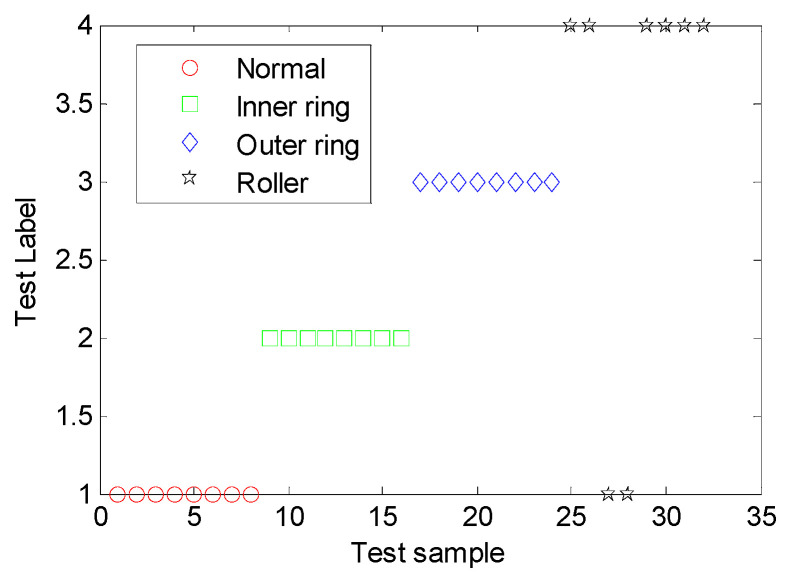
Diagnostic results of testing sample on inter-shaft bearing faults.

To support the generalization ability of the MDERF model, the testing samples of 32 groups were adopted to validate the model by classification. The results are shown in Figure 12 and Table 3.

As revealed in Table 3 and Figure 12, the recognition precision of the built MDERF model is 93.75%. It accurately recognizes the testing samples for inner race fault, outer race fault, and normal statues. However, for eight ball fault samples, two samples were mistakenly recognized as outer race fault samples. The information exergy is the disturbance degree of bearing failure information. From Table 1, the information entropy point vector of the ball fault and outer ring fault is very similar, namely, the disorder degree of the two-failure information is similar. This is the reason for the miscalculation. Therefore, the MDERF model is validated to have good generalization ability. The proposed MDERF model provides a new way for inter-shaft bearing fault diagnosis.

### 5.5. Method Validation

To verify the effectiveness of the developed MDERF model in inter-shaft bearing fault diagnosis, five fault diagnosis algorithms, i.e., support vector machine (SVM) [8,27], k-nearest neighbor (KNN) [28], classification and regression tree (CART) [29], and gradient boosting decision-tree (GBDT) [30], MDERF, are validated by learning and testing with the same test samples. The performance comparison results of five fault diagnosis algorithms based on test set data are shown in Table 4.

As seen from Table 4, the decision-tree diagnosis algorithms, represented by CART, RF, and GBDT, are significantly better in diagnostic accuracy than the distance discrimination algorithms such as SVM and KNN. What is more, the accuracy of the RF and GBDT algorithms through the integrated training of decision-trees is higher than the single decision-tree represented by CART. It is demonstrated that the developed MDERF method is accurate (93.75%) in inter-shaft bearing fault diagnosis.

## 6. Conclusions

The objective of this paper is to propose a novel fault diagnosis method of inter-shaft bearing, i.e., multi-domain entropy-random forest (MDERF) method, by fusing the multi-domain entropy and random forest methods, to improve the precision of fault diagnosis. We discuss the theory and method of MDERF with an emphasis on four information entropies (singular spectrum entropy (SSE), power spectrum entropy (PSE), wavelet energy spectrum entropy (WESE), and wavelet space feature spectrum entropy (WSFSE)) and the random forest method. Then, the developed method is applied to the fault diagnosis of inter-shaft bearing. Through the comparison of methods, the developed MDERF method is validated to be effective and accurate. The results from this study demonstrate that; (1) the fault samples comprising four information entropies have good separability and are suitable for the expression of fault features; (2) the MDERF model is effective to inter-shaft bearing faults diagnosis by adopting the AE signal; (3) the MDERF model is validated to have good learning ability and generalization ability with the diagnostic precision 93.75% and no overfit phenomenon. The efforts of this study provide a new useful insight for inter-shaft bearing fault diagnosis. The proposed method will be extended to multi-faults, and an experimental study on multi-faults of inter-shaft bearings will be carried out to verify the effectiveness of the method.

## Figures and Tables

**Table 1:** Selected fault samples with MDEP.

Fault Type	Sample Number	Vector of Multi-Domain Entropy Point							Type No.
Normal	1	51.22	52.18	52.03	53.28	53.22	52.93	52.75	1
		53.29	53.32	53.67	53.56	54.07	53.66		
	2	51.36	52.55	52.40	53.30	53.22	53.04	52.80	1
		53.08	53.26	53.89	53.32	53.65	54.02		
Inner ring fault	1	51.57	52.30	52.18	51.27	51.72	51.54	51.41	2
		51.99	53.11	51.67	52.39	52.08	53.37		
	2	51.35	51.47	51.13	51.14	51.43	51.04	51.88	2
		50.88	51.99	51.10	51.87	52.65	52.93		
Outer ring fault	1	52.85	53.17	51.50	52.12	52.14	52.30	52.54	3
		52.13	52.40	52.04	52.44	52.59	53.26		
	2	52.93	52.47	51.78	52.36	52.60	51.98	52.76	3
		52.37	52.06	51.84	52.34	52.64	52.94		
Roller fault	1	52.84	53.49	52.54	51.97	52.92	52.45	52.48	4
		52.45	52.35	52.06	52.72	53.24	53.03		
	2	53.19	53.04	52.89	52.13	52.53	52.69	52.51	4
		52.53	52.90	52.29	52.57	52.90	53.08		

**Table 2:** Diagnostic results of training sample on inter-shaft bearing faults.

Confusion Matrix		Real				Precision
		Normal	Inner Ring Fault	Outer Ring Fault	Roller Fault	
Predict	Normal	10	0	0	0	100%
	Inner ring fault	0	10	0	0	
	Outer ring fault	0	0	10	0	
	Roller fault	0	0	0	10	

**Table 3:** Diagnostic results of testing sample on inter-shaft bearing faults.

Confusion Matrix		Real				Precision
		Normal	Inner Ring Fault	Outer Ring Fault	Roller Fault	
Predict	Normal	8	0	0	2	93.75%
	Inner ring fault	0	8	0	0	
	Outer ring fault	0	0	8	0	
	Roller fault	0	0	0	6	

**Table 4:** Comparison of different algorithms in diagnostic precision.

Algorithm	Precision
SVM	80.45%
KNN	78.25%
CART	85.46%
GBDT	87.23%
RF	93.75%
